# The Effect of the Glucosinolate Sinigrin on Alterations in Molecular Biomarkers of the Myocardium in Swiss Mice

**DOI:** 10.3390/foods14020327

**Published:** 2025-01-20

**Authors:** Nikola Ferara, Vedran Balta, Domagoj Đikić, Dyana Odeh, Ana Mojsović-Ćuić, Lana Feher Turković, Dario Dilber, Anđelo Beletić, Irena Landeka Jurčević, Ivana Šola

**Affiliations:** 1 Department of Dermatology and Venereology, Sestre Milosrdnice University Hospital Centre, Vinogradska cesta 29, 10000 Zagreb, Croatia; nfera94@gmail.com; 2 Faculty of Science, University of Zagreb, Rooseveltov trg 6, 10000 Zagreb, Croatia; 3 School of Applied Health Sciences, University of Zagreb, Mlinarska cesta 38, 10000 Zagreb, Croatia; 4 Department of Cardiology, Thalassotherapia Opatija, Maršala Tita 188, 51410 Opatija, Croatia; dario.dilber@gmail.com; 5 Laboratory of Proteomics, Internal Diseases Clinic, Faculty of Veterinary Medicine, University of Zagreb, Heinzelova 55, 10000 Zagreb, Croatia; 6 Faculty of Food Technology and Biotechnology, University of Zagreb, Pierottijeva 6, 10000 Zagreb, Croatia

**Keywords:** glucosinolate, sinigrin (allyl-glucosinolate), myocard, gender, cardiac physiology

## Abstract

Glucosinolates are chemically stable compounds that exhibit biological activity in the body following hydrolysis catalyzed by the enzyme myrosinase. While existing *in vitro* and *in vivo* studies suggest that the hydrolysis products of glucosinolates predominantly exert beneficial effects in both human and animal organisms, some studies have found that the excessive consumption of glucosinolates may lead to toxic and anti-nutritional effects. Given that glucosinolates are primarily ingested in the human diet through dietary supplements and commercially available cruciferous vegetables, we investigated the *in vivo* effects of the glucosinolate sinigrin on molecular markers in the myocardia of healthy Swiss mice. This study aims to elucidate whether sinigrin induces positive or negative physiological effects in mammals following consumption. The alterations in myocardial parameters were assessed by measuring metabolic, inflammatory, structural, and antioxidant markers. Our findings revealed that subchronic exposure to sinigrin in the myocardia of female mice resulted in a significant increase (*p* ≤ 0.05) in the levels of the myokine irisin, matrix metalloproteinases (MMP-2, MMP-9), catalase (CAT), and total glutathione (tGSH), alongside a marked decrease (*p* ≤ 0.05) in the levels of atrial natriuretic peptide (ANP), compared to the control group consisting of both female and male mice. These results suggest that the hydrolysis products of sinigrin may exert a potentially toxic effect on the myocardial tissue of female mice and possess the capability to modulate transcription factors *in vivo* in a sex-dependent manner. This observation calls for further investigation into the mechanisms regulating the actions of glucosinolate hydrolysis products, their interactions with sex hormones, and the determination of permissible intake levels associated with both beneficial and adverse outcomes.

## 1. Introduction

Contemporary lifestyle and dietary patterns, characterized by a low consumption of fruits and vegetables in many developed countries, contribute to stress and negatively affect human health, creating a need for studies focused on maintaining and improving human well-being through dietary habits. Increasing evidence suggests that the consumption of fruits and vegetables aids in the prevention of various diseases due to them containing numerous chemical compounds that have a positive impact on the health of both humans and animals [1]. In this context, considerable attention is given to glucosinolates, a group of secondary metabolites that can predominantly be found in the young parts of widely cultivated and commercially available plant species from the *Brassicaceae* family, which includes vegetables such as cabbage, broccoli, cauliflower, kale, brussels sprouts, and canola [2,3,4]. It is important to emphasize that glucosinolates are predominantly physiologically inactive compounds that are converted into biologically active products following degradation, which is mediated by the enzyme myrosinase, primarily isothiocyanates with potential health benefits [5]. Numerous epidemiological studies confirm a correlation between the consumption of vegetables from this family and a reduced risk of cardiometabolic and neurological diseases, cancers, musculoskeletal disorders, and the development of other conditions associated with oxidative stress [1].

Although a total of 130 glucosinolates have been identified to date, only 5 glucosinolates are widely present in the human diet, including glucobrassicin, sinigrin, glucoerucin, glucoraphanin, and glucoiberin [6,7]. The levels of glucosinolates ingested depend on the available variety of vegetables, agronomic factors, and both the storage and processing of the vegetables prior to their consumption [8]. One of the most studied and prevalent glucosinolates in the *Brassicaceae* family is sinigrin (allyl glucosinolate or 2-propenyl glucosinolate). The main food sources of sinigrin in human nutrition, as well as its highest contents per 100 g of fresh weight of appropriate vegetables, can be found in broccoli (0.19 mg), brussels sprouts (44.50 mg), savoy (17.06 mg), red cabbage onion (3.77 mg), white cabbages (16.31 mg), cauliflower (4.74 mg), and kale (12.47 mg). It has been reported that *Brassicaceae juncea* L. (Indian mustard) and *Sinapis alba* L. contain significant amounts of sinigrin and, after pressing the seeds for oil, the remaining seed meal contains sinigrin concentrations up to 10% by weight [9,10]. Sinigrin is hydrolyzed to allyl isothiocyanate by plant myrosinase and a range of Gram-positive intestinal flora, including *Lactobacillus agilis*, *Streptomyces*, *Bacillus*, and *Staphylococcus* spp. [11] A recent study on rats indicates that allyl isothiocyanate (AITC), a product of sinigrin hydrolysis, induces apoptosis and inhibits the proliferation of liver tumor cells through enzyme regulation without causing any toxicity to the liver. Numerous other studies also indicate the positive effects of sinigrin in preventing the growth and development of tumors of the tongue, esophagus, small and large intestines, breast, and urinary bladder [9,12,13]. Additional beneficial properties of sinigrin pertain to cardiometabolic disorders such as dyslipidemia, insulin resistance, hypertension, impaired glucose tolerance, and central adiposity. It has been established that the intake of glucosinolates in animal models of rodents fed with high-fat diets contributes to reductions in total serum cholesterol, LDL cholesterol, inflammatory cytokines, and the progression of atherosclerotic lesions and hypertension [2]. In a human study, the consumption of 100 g of broccoli sprouts was shown to increase HDL levels and reduce total cholesterol compared to baseline values. Positive effects have also been observed on blood pressure in animal models. A recent study found that daily feeding of dried broccoli sprouts for four months resulted in decreased blood pressure in rats predisposed to spontaneous hypertensive strokes. However, it is still not fully established whether the improvements in cardiometabolic diseases related to the consumption of *Brassicaceae* vegetables are only short-term benefits; thus, further research on this topic is essential [1].

Despite a significant portion of *in vivo* and *in vitro* studies indicating the positive effects of glucosinolates, some studies point out that the excessive consumption of cruciferous vegetables leads to an excessive intake of glucosinolates, which can then be converted into toxic products through the action of the enzyme myrosinase, subsequently leading to the development of undesirable toxic and anti-nutritional effects. Some toxic effects of these compounds include the following: goiter due to impaired iodine availability caused by isothiocyanates; toxic effects on liver and kidney function associated with nitriles; stunted growth in animals, along with toxic effects on the liver and thyroid gland, caused by goitrin; inhibition of thyroid function leading to atrophy and goiter associated with oxazolidin-2-thione [14]. Additionally, recent studies have indicated that AITC may cause bradycardia, atrioventricular block, or abnormal electrocardiograms [2]. Conversely, the consumption of sulforaphane-rich broccoli over a two-week period has been linked to normalized heart rates and improved left ventricular function in a rat model of arrhythmia induced by cardiac stress [2]. However, in a particular group of subjects, it was observed that sulforaphane may induce damage; thus, it is not recommended as a dietary supplement for obesity reduction in heart disease patients. This indicates that each of the hydrolysis products of glucosinolates has its own unique and probably dual properties, suggesting that certain compounds within the glucosinolate group can possess anti-cancer effects, while also exhibiting genotoxic and potentially carcinogenic activities [15]. To prevent such negative effects, efforts are being made to inactivate the myrosinase enzyme through pre-treatment, before the actual extraction of glucosinolates, in order to reduce the presence of possibly harmful products. Given the evidence of possible negative effects, it is necessary to monitor the quantity of glucosinolates consumed and, consequently, their degradation products to protect one’s personal health. Due to insufficient data on the permissible doses of glucosinolates associated with positive or negative effects, further research is necessary to mitigate the harmful effects of glucosinolate hydrolysis [16]. In light of all the above, this study investigated the *in vivo* effect of the commercial glucosinolate sinigrin on molecular markers of the myocardium in healthy Swiss mice of both sexes by monitoringtheir metabolic, inflammatory, structural, and antioxidant changes of the myocardia Additionally, the aim was to assess whether sinigrin induces positive or negative effects on the physiology of mammals following its consumption.

## 2. Materials and Methods

### 2.1. Chemicals

In this study, commercial analytical grade sinigrin (purity: >98%, Sigma-Aldrich, St. Louis, MO, USA) and a 0.9% sodium chloride solution (B. Braun Adria d. o. o., Zagreb, Croatia) were used.

### 2.2. Sinigrin Solution

Aqueous solution of commercial sinigrin (Sigma-Aldrich, St. Louis, MO, USA) was prepared by dissolving sinigrin in purified water (aqua pro) (B. Braun Adria d. o. o., Zagreb, Croatia) at a dose of 10 mg/kg of body weight of the animal. The dose in this study was selected based on previous studies and from the consumption amount of *Brassica* vegetables used by a clinical trial [9,17,18,19,20].

### 2.3. Animals and Ethics Statement

This study was conducted on mice from the laboratory animal breeding unit of the Department of Animal Physiology at the Faculty of Science, University of Zagreb, Croatia. The experimental subjects were Swiss albino mice aged 3 months. A total of 24 animals of both sexes were included in this study, comprising 12 females and 12 males. From the beginning of this study, the animals were housed under standard conditions (6 mice per cage, 12 h of light/12 h of dark at 22 °C and 60% humidity) with access to food and water *ad libitum*. The mice were fed a standard mouse diet (standard diet GLP, 4RF21, Mucedola, Settimo Milanese MI, Milan, Italy). The experiments were approved by the Ethics Committee of the Faculty of Science, University of Zagreb, Croatia (approval code: 251-58-10617-14-21, date of approval: 13 January 2021). The maintenance and care of all experimental animals were conducted in accordance with the regulations in force in the Republic of Croatia (Animal Protection Act, NN 102/2017 [21]; Amendments to the Animal Protection Act, NN 37/13 [22]; Regulation on the Protection of Animals Used for Scientific Purposes, NN 55/13 [23]) and according to the Guide for the Care and Use of Laboratory Animals, DHHS (NIH) Publ # 86-23, National Research Council [24].

### 2.4. Experimental Design

Before the experiment commenced and throughout this study, the animals were individually marked and weighed (using a digital scale ABS 220-4, Kern Sohn, Balingen, Germany), and were assigned to experimental groups based on sex and aiming to achieve a similar body weight distribution (body weight 25 ± 2 g). The quantity of individual preparations administered during the experiment was determined based on the body weights of the animals. During the course of the experiment, the animals received individual preparations orally via intragastric (*ig*) administration once daily, for 28 days, using a gastric cannula. The groups and treatment methods were as follows:

- Cont._F_—female control group (0.3 mL of physiological saline);
- Cont._M_—male control group (0.3 mL of physiological saline);
- Sinig._F_—females treated with an aqueous solution of sinigrin (0.3 mL, 10 mg/kg of body weight per day);
- Sinig._M_—males treated with an aqueous solution of sinigrin (0.3 mL, 10 mg/kg of body weight per day).

The animals were euthanized after 28 days of the experiment. During the euthanasia procedure, all animals were adequately anesthetized and analgesized via intraperitoneal (*ip*) administration of a combination of Narketan^®^ Vetoquinol S.A., BP 189 Lure Cedex, France (active substance: ketamine) and Xylapana^®^ Vetoquinol Biowet Sp., Gorzow Wielkopolski, Poland (active substance: xylazine) at a dose of 25 mg/kg of body weight, and myocardium was collected for further analyses.

### 2.5. Preparation of Cardiac Tissue Homogenate for Biomarker Measurements

After isolating the heart, the cardiac tissue was rinsed in pre-chilled phosphate buffer to remove any residual blood and then weighed on an analytical scale. To determine metabolic, structural, anti-inflammatory, and antioxidant parameters in the cardiac tissue, the cardiac tissue was diced into smaller pieces and then homogenized in phosphate buffer at a ratio of 1:10. Cardiac tissue samples were sonicated using an ultrasonic homogenizer (Bandelin Sonoplus HD2070, Bandelin, Berlin, Germany) with an MS73 probe, at a power setting of 10%. The samples were sonicated in three cycles of 30 s each, with a 10 s pause between cycles, at +4 °C. All procedures were performed on ice. The sonicated homogenates were centrifuged at 20,000× *g* for 15 min in a Mikro 200R ultracentrifuge (Hettich, Kirchlengern, Germany) with cooling at +4 °C. The resulting supernatants were used for immediate biomarker measurement or stored at −80 °C for further analysis.

### 2.6. Determination of Protein Concentration

The protein concentration in the cardiac tissue homogenates was determined using the Lowry method [25] and expressed in milligrams of protein per milliliter (mg/mL). Bovine serum albumin (BSA) was used as the standard. The protein concentration in the tissue samples was utilized to express the values of the measured parameters in the cardiac tissue homogenates.

### 2.7. Determination of Metabolic Parameters of the Myocardium by Measuring the Concentration of Adropin, Irisin, Atrial Natriuretic Peptide (ANP), and Peroxisomal Acyl-CoA Oxidase 1 (ACOX1)

The concentrations of adropin, irisin, and ANP were determined using the Mouse Adropin ELISA Kit, Mouse Irisin ELISA Kit, and Mouse ANP ELISA Kit from ELK Biotechnology Co., Ltd. (Denver, CO, USA). The concentration of ACOX1 was measured using the Mouse ELISA Kit ACOX1 from MyBioSource (San Diego, CA, USA). All measurements were performed according to the manufacturer’s instructions in cardiac tissue homogenates diluted with the appropriate dilution buffer. The intensity of the color change was proportional to the concentrations of adropin, irisin, ANP, and ACOX1, and absorbance values were read at a wavelength of 450 ± 10 nm using a Tecan Infinite 200 microplate reader (Tecan, Hamburg, Germany). Based on standard curves relating absorbance to the concentration of standard solutions of adropin, irisin, ANP, and ACOX1, linear regression equations were determined, and the concentrations of adropin and ACOX1 in heart tissue were expressed in ng/mg of protein, while those of irisin and ANP were expressed in pg/mg of protein. All assays were performed and measured in duplicate, and the dilution factor was considered in the final concentration calculation.

### 2.8. Determination of Structural Parameters of the Myocardium by Measuring the Concetrations of High-Sensitivity Cardiac Troponin hs-cTnI)

Hs-cTnI concentration was measured using a UniCel Dxl 600 immunoassay system (Beckman Coulter, Indianapolis, IN 46268, USA). This chemiluminescent method is based on luminescent oxygen channeling immunoassay (LOCI) technology. The LOCI reagents include two synthetic microsphere reagents: streptavidin-coated microspheres (Sensibeads) containing a photosensitizing dye, and a second type of microsphere (Chemibeads) coated with a different anti-cardiac troponin I monoclonal antibody and containing a chemiluminescent dye. Samples were incubated with Chemibeads and biotinylated anti-cardiac troponin I antibody to form sandwich complexes of microsphere-cardiac TnI-biotinylated antibody. Sensibeads were then added, binding to the biotin to form microsphere pairs. Illumination of the complexes at 680 nm initiated a chemiluminescent reaction, and the resulting signal was measured at 612 nm. This signal is directly proportional to the hs-cTnI concentration in the sample. Heart tissue hs-cTnI concentration was expressed as µg/mg protein. All assays were performed and measured in duplicate, with the dilution factor incorporated into the final concentration calculation.

### 2.9. Determination of Inflammatory Parameters in Myocardial Tissue by Measuring the Concentration of Matrix Metalloproteinases 2 and 9 (MMP-2, MMP-9), Pentraxin 3 (PTX-3), and Nitric Oxide (NO)

The concentrations of matrix metalloproteinase 2 (MMP-2), matrix metalloproteinase 9 (MMP-9), and pentraxin 3 (PTX3) were determined using the Mouse MMP-2 ELISA Kit, Mouse MMP-9 ELISA Kit, and Mouse PTX3 ELISA Kit from ELK Biotechnology Co., Ltd. (Denver, CO, USA). All measurements were performed according to the manufacturer’s instructions in cardiac tissue homogenates diluted with the appropriate dilution buffer. The intensity of the color change was proportional to the concentrations of MMP-2, MMP-9, and PTX3, and absorbance values were read at a wavelength of 450 ± 10 nm using a Clariostar plus (BMG Labtech, Offenburg, Germany). Based on standard curves relating absorbance to the concentrations of the standard solutions of MMP-2, MMP-9, and PTX3, linear regression equations were determined, and the concentrations of MMP-2, MMP-9, and PTX3 in cardiac tissue were expressed as ng/mg of protein.

The concentration of nitric oxide (NO) was measured using the spectrophotometric method according to the Griess assay [26]. The Griess reagent system (Promega, Madison, WI, USA) kit was used for the measurement of nitric oxide (II). The Griess reagent system is based on a chemical reaction between sulfanilamide (1% solution of sulfanilamide dissolved in 5% phosphoric acid) and N-1-naphthylethylenediamine dihydrochloride (NED, a 0.1% aqueous solution of N-1-naphthylethylenediamine dihydrochloride) which results in azo-colored compounds. In each well of the microtiter plate, 50 μL of cardiac tissue homogenate and 50 μL of sulfanilamide solution were added, followed by incubation for 10 min at room temperature in the dark. After incubation, 50 μL of NED solution was added to the reaction mixture, and incubation was repeated for an additional 10 min at room temperature in the dark. After incubation (indicated by a visible pink coloration of the sample), the absorbance was measured at 540 nm using a Tecan Infinite 200 microplate reader (Tecan, Hamburg, Germany). A 0.1 M aqueous solution of sodium nitrite was used as the standard, with concentrations ranging from 0 to 100 μM. From the standard curve relating absorbance to nitrite concentration, the slope of the line was determined, and the concentration of nitrite, and consequently NO, was calculated and expressed in μM/protein. All assays were performed and measured in duplicate.

### 2.10. Determination of Oxidative Stress Biomarkers in Myocardial Tissue by Measuring Total Superoxide Dismutase (SOD) Activity, Catalase (CAT) Activity, Lipid Peroxidation, and Total Glutathione (tGSH)

The total activity of superoxide dismutase (SOD) was determined using the Mouse T-SOD ELISA Kit from MyBioSource (San Diego, CA, USA). The measurement was conducted according to the manufacturer’s instructions based on the WST-1 (Water-soluble tetrazolium salt-1) method, in which xanthine oxidase (XO) catalyzes a chemical reaction between WST-1 and the superoxide anion, resulting in the formation of a blue-colored WST-1 formazan. The SOD inhibits this reaction by reacting with the superoxide anion, allowing for the measurement of SOD activity through absorbance readings. The activity of SOD is inversely proportional to the development of color. Absorbance of the samples was measured at 440 nm using a Tecan Infinite 200 microplate reader (Tecan, Hamburg, Germany), and the results were expressed as U/mg of protein.

Catalase (CAT) activity in tissues was assayed by measuring the initial rate of H_2_O_2_ disappearance at 240 nm with Libro S22 spectrophotometer (Biochrom, Cambridge, UK) [27]. Catalase activity was calculated using the extinction coefficient of H_2_O_2_ (ε = 39.4 mM^−1^ cm^−1^), and the results of catalase activity were expressed as U/mg of protein.

Lipid peroxidation was determined by measuring the concentration of MDA using a modified version of the method of Ohkawa et al. [28] for the detection of thiobarbiturate reactive species (TBARSs), whose concentration was measured spectrophotometrically (Libra S22, Biochrom, Cambridge, UK) at a wavelength of 532 nm. The MDA levels were determined using the molar absorption coefficient for the malondialdehyde–thiobarbiturate (MDA–thiobarbituric acid, TBA) complex, which is 1.56 × 10^5^ M^−1^ cm^−1^. The results of the measurements were expressed in nmol/mg of protein.

The concentration of total glutathione (tGSH) was determined using a modified version of the method described by Tietze [29]. The procedure for measuring total glutathione concentration is based on the chemical reaction between the thiol reagent 5,5′-dithiobis-2-nitrobenzoic acid (DTNB, Ellman’s reagent) and GSH, resulting in the formation of chromophore 2-nitro-5-thiobenzoic acid (NTB) and a small amount of glutathione disulfide (GSSG). The absorbance of the samples was measured at 440 nm using a Tecan Infinite 200 microplate reader (Tecan, Hamburg, Germany), and the concentration of tGSH was calculated using the molar absorption coefficient ε(DTNB) = 8.22 M^−1^ cm^−1^. The concentration of tGSH was expressed as mU/mg of protein.

### 2.11. Statistical Analysis

Statistical analysis was performed using the STATISTICA 14 program (StatSoft, Tulsa, OK, USA) [30]. All data were presented as mean ± standard error (mean ± SE). The data were analyzed using the Kruskal–Wallis nonparametric test and were considered significant at the level of *p* < 0.05. Further analysis of the differences between the groups was made by multiple comparisons of the mean values of all groups. All data are represented using GraphPad Prism 7 software (GraphPad Software, Inc., La Jolla, CA, USA) [31].

## 3. Results

### 3.1. Metabolic Parameters

**Figure 1:**
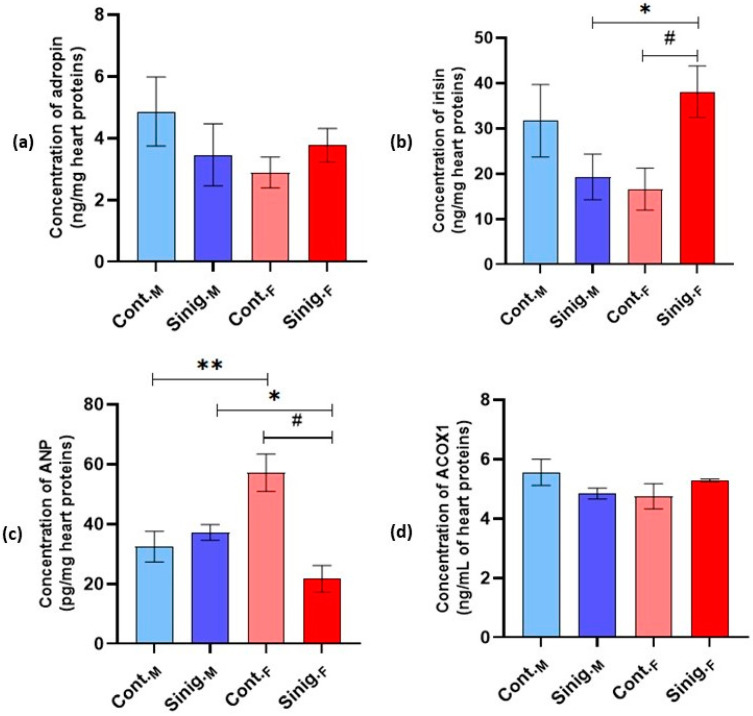
The effect of sinigrin on the concentrations of (**a**) adropin, (**b**) irisin, (**c**) atrial natriuretic peptide (ANP), and (**d**) peroxisomal acyl-CoA oxidase I (ACOX1) in cardiac tissue homogenates. Mice (N = 6) in both female and male control groups were treated intragastrically (*ig*) with 0.3 mL of physiological saline, while in the experimental groups, females and males were treated *ig* with 0.3 mL of an aqueous solution of sinigrin at a dose of 10 mg/kg once daily for 28 days. #: statistically significantly different from the Cont.F group (*p* ≤ 0.05); *: statistically significantly different from the Sinig._M_ group (*p* ≤ 0.05); **: statistically significantly different from the Cont._M_ group (*p* ≤ 0.05). The results are presented as the mean ± standard error. Abbreviations: Cont._M_—male control group; Cont._F_—female control group; Sinig._M_—group of males treated with an aqueous solution of sinigrin; Sinig._F_—group of females treated with an aqueous solution of sinigrin.

Analysis of the metabolic parameters in the myocardia of mice revealed a statistically significant increase in the concentration of irisin in the females treated with an aqueous solution of sinigrin (Sinig._F_), compared to the female control group (Cont._F_) and the group of males treated with the aqueous solution of sinigrin (Sinig._M_) (*p* ≤ 0.05) (Figure 1b). In the analysis of the atrial natriuretic peptide (ANP) concentrations, a statistically significant higher concentration was found in the female control group (Cont._F_) compared to the male control group (Cont._M_) and both the females and males treated with the aqueous solution of sinigrin (Sinig._F_, Sinig._M_) (Figure 1c). No statistically significant differences (*p* ≤ 0.05) were observed in the concentrations of adropin and peroxisomal acyl-CoA oxidase I (ACOX1) between the examined groups (Figure 1a,d).

### 3.2. Structural Parameters—High-Sensitivity Cardiac Troponin I (hs-cTnI)

**Figure 2:**
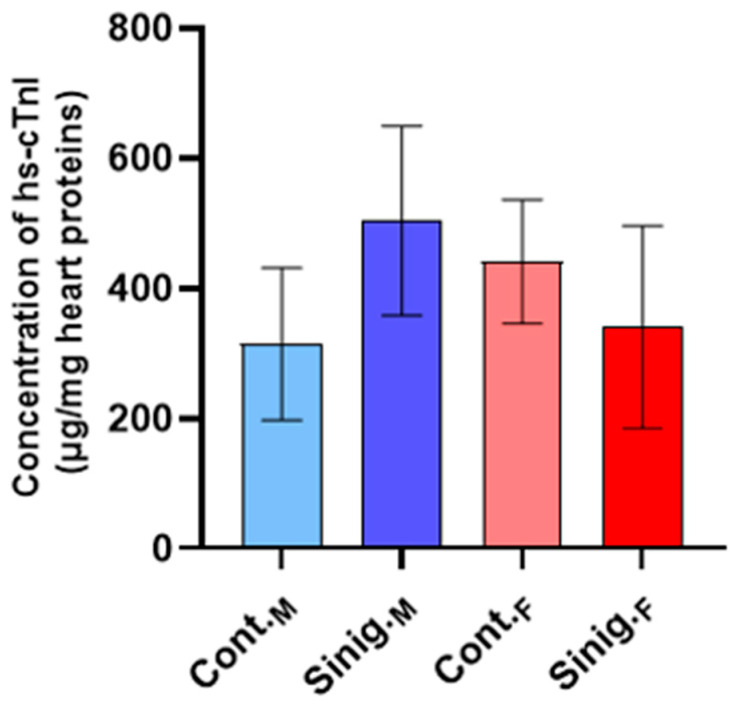
The effect of sinigrin on the concentration of high-sensitivity cardiac troponin I (hs-cTnI) in cardiac tissue homogenates. Mice (N = 6) in both the female and male control groups were treated intragastrically (*ig*) with 0.3 mL of physiological saline, while in the experimental groups, females and males were treated *ig* with 0.3 mL of an aqueous solution of sinigrin at a dose of 10 mg/kg once daily for 28 days. The results are presented as the mean ± standard error. Abbreviations: Cont._M_—male control group; Cont._F_—female control group; Sinig._M_—experimental group of males treated with an aqueous solution of sinigrin; Sinig._F_—experimental group of females treated with an aqueous solution of sinigrin.

Analysis of the concentrations of high-sensitivity cardiac troponin I (hs-cTnI) in the cardiac tissue homogenates between the control and experimental groups of males and females revealed no statistically significant differences (*p* ≤ 0.05) (Figure 2).

### 3.3. Inflammatory Parameters

**Figure 3:**
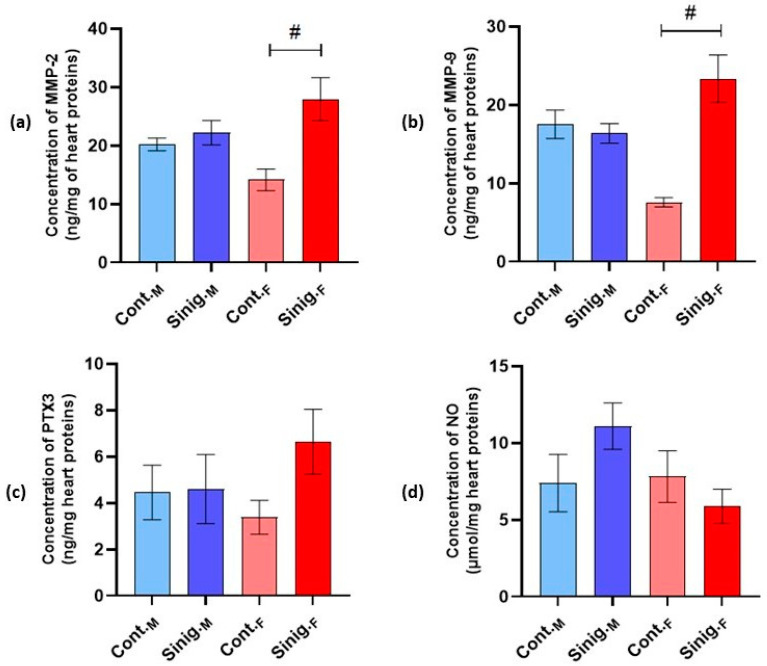
The effect of sinigrin on the concentrations of (**a**) matrix metalloproteinase 2 (MMP-2), (**b**) matrix metalloproteinase 9 (MMP-9), (**c**) pentraxin 3 (PTX3), and (**d**) nitric oxide (NO) in cardiac tissue homogenates. Mice (N = 6) in both the female and male control groups were treated intragastrically (*ig*) with 0.3 mL of physiological saline, while in the experimental groups, females and males were treated *ig* with 0.3 mL of an aqueous solution of sinigrin at a dose of 10 mg/kg once daily for 28 days. #: statistically significantly different from the Cont._F_ group (*p* ≤ 0.05). The results are presented as the mean ± standard error. Abbreviations: Cont._M_—male control group; Cont._F_—female control group; Sinig._M_—experimental group of males treated with an aqueous solution of sinigrin; Sinig._F_—experimental group of females treated with an aqueous solution of sinigrin.

The analysis of inflammatory biomarkers in cardiac tissue homogenates revealed a statistically significant increase in the concentrations of matrix metalloproteinases 2 (MMP-2) and 9 (MMP-9) in the females treated with an aqueous solution of sinigrin (Sinig._F_) compared to the control group of females (Cont._F_) (*p* ≤ 0.05) (Figure 3a,b). In the analysis of the pentraxin 3 (PTX3) and nitric oxide (NO) concentrations, no statistically significant differences were observed between the groups (*p* ≤ 0.05).

### 3.4. Oxidative Stress Biomarkers

**Figure 4:**
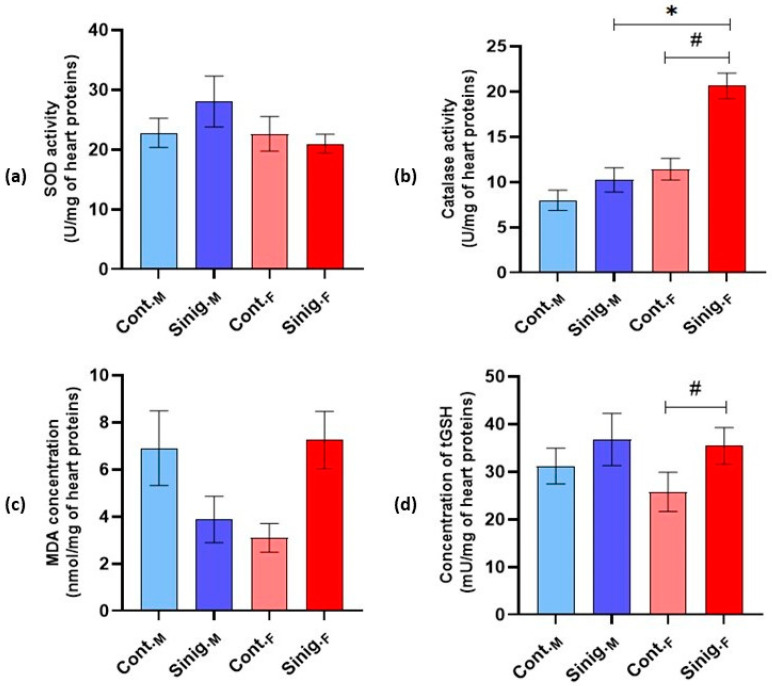
The effect of sinigrin on (**a**) superoxide dismutase (SOD) activity, (**b**) catalase (CAT) activity, (**c**) malondialdehyde (MDA) concentration, and (**d**) total glutathione (tGSH) concentration in cardiac tissue homogenates. Mice (N = 6) in both the female and male control groups were treated intragastrically (*ig*) with 0.3 mL of physiological saline, while in the experimental groups, females and males were treated *ig* with 0.3 mL of an aqueous solution of sinigrin at a dose of 10 mg/kg once daily for 28 days. #: statistically significantly different from the Cont.F group (*p* ≤ 0.05); *: statistically significantly different from the Sinig._M_ group (*p* ≤ 0.05). The results are presented as the mean ± standard error. Abbreviations: Cont._M_—male control group; Cont._F_—female control group; Sinig._M_—experimental group of males treated with an aqueous solution of sinigrin; Sinig._F_—experimental group of females treated with an aqueous solution of sinigrin.

The analysis of oxidative stress biomarkers in cardiac tissue homogenates revealed a statistically significant increase in total glutathione (tGSH) concentration in the females treated with an aqueous solution of sinigrin (Sinig._F_) compared to the control group of females (Cont._F_). Also, the catalase (CAT) activity in the females treated with an aqueous solution of sinigrin (Sinig._F_) was significantly higher in comparison to both the control group of females (Cont._F_) and that of males treated with an aqueous solution of sinigrin (Sinig._M_) (*p* ≤ 0.05) (Figure 4b,d).

## 4. Discussion

Almost a quarter of all pharmaceutical products worldwide are derived from plants. Recently, significant attention has been given to glucosinolates as potential therapeutics for the prevention and treatment of various diseases, including cancer, diabetes, and hypertension [32]. However, although they exert numerous positive effects on human health, scientific research indicates that certain hydrolysis products of glucosinolates, particularly isothiocyanates, can exhibit significantly different properties and effects on health, including toxic ones [15,33,34,35]. For this reason, extensive research is necessary to gain a detailed understanding of their mechanisms of action *in vivo* before implementing glucosinolates and their hydrolysis products as potential targeted therapeutics.

Current knowledge says that microbial myrosinase (β-thioglucoside glucohydrolase) in the digestive systems of humans and mice can hydrolyze sinigrin into isothiocyanates [36,37,38], specifically AITC, which is the primary hydrolysis product of sinigrin. However, the hydrolysis process is complex and can yield a range of products besides AITC, depending on factors such as the pH, temperature, enzyme activity (myrosinase), and presence of other compounds. Generally, through unstable intermediates, thiohydroxamate-O-sulfonates, glucosinolates can be converted into isothiocyanates, thiocyanates, nitriles, or epithionitriles [39]. While isothiocyanates, the most-studied hydrolysis products, are formed via spontaneous Lossen rearrangement of the corresponding aglucone under physiological conditions, the presence of so-called specifier proteins (group of kelch domain-containing proteins), low pH conditions, and an abundance of ferrous ions (Fe^2+^) favor the formation of non-isothiocyanates products. It is presently assumed that specifier proteins interact with myrosinase to capture the glucosinolate aglucone before its spontaneous conversion to an isothiocyanate [40]. It is worth mentioning that sinigrin is the only known glucosinalte that can form isothiocyanates alongside other products such as nitriles, epithionitriles, and thiocyanates [5]. The isothiocyanates have been shown to possess cardioprotective, antioxidant, anti-inflammatory, neuroprotective, and antimicrobial properties, without demonstrating any significant negative effects [35,41], while nitriles and epithionitriles were shown to have less health-protective potential [42]. In comparison to isothiocyanates, both nitriles and epithionitriles show reduced effects of inducing phase II detoxification enzymes and there is substantial evidence of them having both cytotoxic and genotoxic effects. Since it is impossible to precisely determine the exact mechanism of sinigrin conversion and all its hydrolysis products, it is important to remember that the observed effects of administered sinigrin cannot be solely attributed to AITC. Other hydrolysis products may also play a role, and some of these may have toxic effects in addition to any protective effects. It is reasonable to assume that significant conversion of sinigrin to isothiocyanates (ITCs) occurs under physiological conditions [43], but the extent of this conversion is influenced by many factors, including the type of *Brassica* plant, the growing conditions, and the preparation methods [40]. While our research primarily focused on the effects of AITC as the presumed main hydrolysis product of sinigrin, we must not overlook the potential effect of other hydrolysis products, even though their effects on health may be less prominent and therefore less extensively studied.

One of the aspects of the cardioprotective effects of isothiocyanates is attributed to their ability to donate hydrogen sulfide (H_2_S), which has attracted the interest of many researchers in understanding the pharmacological and nutraceutical properties of isothiocyanates. However, to date, only a few researchers have demonstrated that these natural sulfur compounds can induce beneficial cardiovascular effects, such as vasorelaxation and antihypertensive effects, which are linked to their ability to release H_2_S in biological tissues [44]. It is known that isothiocyanates form adducts with cysteine that intramolecularly cyclize, releasing H_2_S, raphanusanine acid, organic amines, and dihydrothiazole derivatives [45,46]. It is important to note that some researchers have found that glucosinolates themselves, in their native form (without prior conversion to isothiocyanates), can release H_2_S through a cysteine-dependent mechanism, which grants them biological activity and may, in the future, change the prevailing view of their biological inertness [47].

Testai et al. [48] investigated the efficacy of 4-carboxyphenyl isothiocyanate as an exogenous H_2_S donor in cardioprotection against ischemia/reperfusion (I/R) injury in the hearts of rats and mice. In Langendorff-perfused rat hearts subjected to I/R, 4-carboxyphenyl isothiocyanate significantly improved the post-ischemic recovery of functional myocardial parameters and limited tissue damage. These effects were antagonized by 5-hydroxydodecanoic acid (a blocker of mitoKATP channels). Additionally, 4-carboxyphenyl isothiocyanate inhibited the formation of reactive oxygen species. In mouse hearts, 4-carboxyphenyl isothiocyanate reduced the post-ischemic release of norepinephrine and the incidence of ventricular arrhythmias. Based on this research, the authors concluded that H_2_S-releasing compounds based on isothiocyanate, such as 4-carboxyphenyl isothiocyanate, may be considered a suitable pharmacological option in anti-ischemic therapy.

Furthermore, Harvey et al. [49] explored the antioxidant effects and potential H_2_S-releasing capacity of the glucosinolates glucoraphasatin, glucosinolate, and glucohesperin in rat cardiomyocytes. Their findings indicated that the tested glucosinolates did not affect the viability of cardiac cells, but exhibited the ability to protect cardiac cells from hydrogen peroxide-induced oxidative stress and apoptosis. Neither pure glucosinolates nor those mixed with cysteine, N-acetylcysteine, glutathione, H_2_O_2_, iron, pyridoxal-5′-phosphate, or mouse liver lysates induced H_2_S release. The addition of glucosinolates also did not alter the endogenous H_2_S concentrations in cardiac cells, while H_2_O_2_ significantly induced the oxidation of cysteine in cystathionine γ-lyase protein and inhibited the rate of H_2_S production. The researchers concluded that the tested glucosinolates protect cardiomyocytes from oxidative stress and apoptosis, but that they did so independently of H_2_S signaling.

On the other hand, Waz and Matouk [50] examined the effect of AITC on the cardiotoxicity of the anthracycline chemotherapy drug doxorubicin (DOX) in male rats, whose toxic effects limit its clinical application. In the group treated with doxorubicin, a mortality rate of 40% and a significant increase in cardiac enzymes were observed, resulting in an increased number of degenerative cardiomyocytes and inflammatory cell infiltrates. In the AITC + DOX group, the application of AITC alleviated oxidative stress in the myocardium induced by DOX, as evidenced by reduced concentrations of malondialdehyde and nitric oxide, alongside increased concentrations of reduced glutathione and superoxide dismutase activity. Additionally, the level of the inflammatory cytokine TNF-α decreased after the administration of AITC with DOX. The cardioprotective effect of AITC was attributed to increased expression of the cytoprotective nuclear factor Nrf2. Moreover, AITC increased the concentration of heme oxygenase 1 (HO-1), which mitigated oxidative stress induced by DOX in the hearts of rats. Based on their findings, the authors concluded that AITC alleviates acute cardiotoxicity associated with doxorubicin treatment by reducing oxidative stress and induced inflammatory tissue injury.

In this study, we investigated the *in vivo* effects of the commercial glucosinolate sinigrin on molecular markers of the myocardium in healthy female and male Swiss mice. This was accomplished by monitoring metabolic, inflammatory, structural, and antioxidant changes to assess its impact after consumption in mammalian organisms. The results for the metabolic parameters in the myocardium indicate a statistically significant increase in the concentration of irisin in females treated with an aqueous solution of sinigrin (Sinig._F_) compared to the control group of females (Cont.F) and the males treated with the aqueous solution of sinigrin (Sinig._M_) (*p* ≤ 0.05) (Figure 1b). A statistically significant higher concentration of atrial natriuretic peptide (ANP) was also found in the female control group (Cont._F_) compared to the male control group (Cont.M) and both the females and males treated with the aqueous solution of sinigrin (Sinig.F, Sinig.M) (Figure 1c).

Irisin is a myokine/adipokine released into the bloodstream through the cleavage of the membrane protein 5, which contains a type III fibronectin domain (FNDC5), and which is triggered by muscle contraction [51]. It is primarily secreted by skeletal and cardiac muscles during exercise [52]. Current research on the role of irisin suggests numerous physiological effects in both human and animal organisms, particularly in regulating a range of endocrine and metabolic functions [53]. For instance, circulating irisin has been shown to increase the expression of mitochondrial uncoupling protein 1 (UCP1), which facilitates the conversion of white adipose tissue into brown adipose tissue [54], and to regulate glucose and lipid homeostasis. Consequently, many studies are focusing on its therapeutic potential in treating metabolic disorders such as obesity and type 2 diabetes [55]. Recent research indicates that irisin dysfunction is likely associated with cardiovascular diseases like hypertension, coronary artery disease, myocardial infarction, and ischemia-reperfusion injury. Additionally, variants of the irisin gene have also been linked to cardiovascular diseases [56].

While the importance of irisin is unquestionable, the results of studies examining the relationship between irisin levels and physical activity are contradictory. For example, the irisin concentrations in patients with type 2 diabetes mellitus are significantly lower compared to those without diabetes, whereas morbidly obese patients exhibit significantly elevated levels of irisin compared to individuals of normal body weight [57].

Current knowledge about irisin suggests that its concentration in the body increases with physical activity. After exercise, skeletal muscles and the myocardium produce irisin along with various cytokines and peptides (IL-6, IL-8, IL-15, fibroblast growth factor (FGF), brain-derived neurotrophic factor (BDNF)). These myokines, together with irisin, modulate energy homeostasis and the effects of exercise on the cardiovascular system. Interestingly, cardiomyocytes produce more irisin than the skeletal muscles [58]. Following myocardial injury, cardiomyocytes initiate cardiac repair processes that are associated with oxidative stress, apoptosis, inflammation, and energy balance, where irisin is believed to play a crucial role. Irisin promotes the cardiac progenitor cells induced regeneration of the myocardium. Additionally, by acting on eNOS signaling, irisin can modulate blood pressure and endothelial dysfunction. However, numerous studies on irisin levels and its prognostic value for cardiovascular diseases have yielded relatively conflicting results, suggesting that irisin may have a dual role in different stages of cardiovascular diseases [58]. Furthermore, the physiological levels of irisin in humans and rodents are influenced by several factors, including physical activity, obesity, diet, disease, and exposure to various medications [59]. Although there is no clear consensus regarding the physiological value of irisin, it is believed that there is a sex dimorphism in irisin levels, with the female sex being independently associated with higher concentrations of irisin, which aligns with our findings [60]. However, research results are inconsistent; while one study reported elevated irisin levels in women and girls compared to men and boys, other studies found no significant differences in irisin levels between genders [61]. Additionally, transient increases in serum irisin after acute exercise have been shown to be more pronounced in lean (“physically fit”) women than in men [59]. There is a growing suspicion that the levels of irisin, as well as its expression in tissues, may be influenced by sex hormone concentrations. Garcés et al. [57] found that the concentration of circulating irisin depends on the levels of female hormones. Their research indicates that, in healthy eumenorrheic women, the irisin concentrations are 26% higher during the luteal phase compared to the follicular phase. Moreover, serum irisin concentrations were found to be higher throughout pregnancy compared to the irisin concentrations during the follicular and luteal phases of the menstrual cycle in non-pregnant women [57]. These results suggest that irisin levels vary depending on the phase of the menstrual cycle. 17β-estradiol (E2) may influence irisin levels by acting on anabolic processes and increasing muscle mass, potentially leading to higher irisin expression and inducing mechanisms of irisin resistance. This is supported by observations that high circulating irisin concentrations correlate with an impaired glucose tolerance, with this correlation being more pronounced in girls than in boys [60]. Furthermore, the answer to the observed sex dimorphism in irisin levels may lie in differences in body fat composition and distribution between genders. Specifically, brown adipose tissue is known to produce irisin, and women, at least in adulthood, tend to have more active brown adipose tissue compared to men. Additionally, irisin is produced by white adipose tissue, and its distribution also shows sexual dimorphism [61]. A study conducted on young and healthy individuals demonstrated that a lean body mass (the value obtained by subtracting fat mass from total body mass) is a strong positive predictor of irisin levels, with these levels being higher in women than in men [60]. Evidence of sex dimorphism in the distribution and effects of irisin has also been observed in research that identified irisin in the central nervous system. Specifically, a study conducted on brain samples from monkeys (marmosets and rhesus monkeys) revealed different distributions of FNDC5 and PGC-1α (peroxisome proliferator-activated receptor-gamma coactivator 1 alpha) based on sex. In female subjects, the pituitary gland and the posterior hypothalamus showed significantly higher transcription levels of FNDC5 and PGC1A compared to male subjects [62].

Given that our study found a statistically significant difference in irisin concentrations between the Sinig._F_ and Sinig._M_ groups, as well as between the Sinig._F_ and Cont._F_ groups, but not between the Cont._M_ and Cont._F_ groups, we cannot fully compare our results with the aforementioned studies.

Parsanathan and Jain [63] investigated the effects of H_2_S deficiency on a decreased secretion of FNDC5 and irisin, as well as glucose metabolism in mice. Mice fed a high-fat diet exhibited elevated blood glucose levels along with significantly reduced levels of H_2_S, PGC-1α, and cystathionine γ-lyase (CSE), the enzyme that produces H_2_S. Additionally, it was found that high-fat-fed mice had reduced levels of FNDC5/irisin and increased oxidative stress parameters in their muscles compared to the control group. High levels of glucose or palmitate reduced the levels of CSE/PGC-1α/FNDC5 and glucose uptake in myotubes. Inhibitors (propargilglycine and aminooxyacetate) of H_2_S-producing enzymes or CSE siRNA significantly decreased the levels of H_2_S and FNDC5 along with PGC-1α; similar H_2_S deficiency conditions also led to decreased GLUT4 expression and glucose uptake. Levels of H_2_S, PGC-1α, and FNDC5, as well as glucose uptake, were significantly elevated after treatment with l-cysteine or an H_2_S donor. The authors suggested that increasing H_2_S levels could have beneficial effects on glucose homeostasis through the activation of the PGC-1α/FNDC5/irisin signaling pathway.

In the context of our research, the statistically significant increase in irisin concentration in female mice treated with an aqueous solution of sinigrin (Sinig._F_) compared to the control group of females (Cont._F_) could potentially be linked to the ability of sinigrin to donate H_2_S, where its increased concentration may have resulted in enhanced activation of the PGC-1α/FNDC5/irisin signaling pathway. However, the nature of this relationship remains unknown, and, thus, we emphasize that this hypothesis is merely indicative, opening potential hypotheses and opportunities for further research.

Analyzing the obtained values of ANP between the experimental groups, it is particularly interesting to point out that female mice treated with an aqueous solution of sinigrin (Sinig._F_) exhibit statistically significantly lower ANP concentrations compared to the control groups of females and males (Cont._F_, Cont._M_), as well as to the males treated with the aqueous solution of sinigrin (Sinig._M_). This result could potentially be interpreted as a reflection of the statistically significant increase in irisin concentration in the myocardia of females treated with the aqueous solution of sinigrin. Specifically, Li et al. [64] proposed that irisin prevents the rise in mRNA ANP levels in the myocardium in response to pressure overload-induced myocardial hypertrophy. Therefore, it is likely that the modulation of ANP production is not a direct effect of irisin, but rather its indirect inhibitory effect on hypertrophy and remodeling. This notion is rather supported by the results from their animal model study of cardiac hypertrophy. Under normal conditions, no significant differences in ANP levels were found among FNDC5 knockout mice (CO), wild-type (WT) mice, or FNDC5 transgenic mice (Tg). In a model of cardiac hypertrophy, induced by transverse aortic constriction (TAC), significantly elevated levels of ANP were found in the hearts of wild-type mice, while even higher levels were observed in FNDC5 knockout mice. In contrast, FNDC5 transgenic mice exhibited significantly reduced ANP levels compared to wild-type mice. This outcome supports the protective role of irisin only in the case of hypertrophic stimuli. While the results of the mentioned study are not entirely aligned with the findings of our research—where a significant decrease in ANP levels was observed specifically in the group with a significantly elevated level of irisin in the myocardium, without the influence of a hypertrophic stimulus—it is important to note that our study involved a group treated with sinigrin, which, aside from its favorable effects, has also been reported to have negative impacts on the myocardium. Similar to hypertrophy in the previously described study, these negative effects could serve as a stimulus for “activating” the protective effect of irisin.

Specifically, for sinigrin, more precisely its active form, AITC, adverse effects have been described when inhaling this compound. In normotensive and spontaneously hypertensive rats, symptoms such as bradycardia, atrioventricular (AV) block, prolonged PR interval, and a biphasic blood pressure response were observed. Additionally, in spontaneously hypertensive rats, AITC caused an abnormal electrocardiogram (ECG) pattern, characterized by an increased occurrence of negative P waves, their prolonged duration, and prolonged PR and RR intervals compared to the control group [2].

Oguri et al. [65] were the first to demonstrate that AITC can activate Ca^2+^-permeable non-selective cation channels in human cardiac fibroblasts, likely acting on TRPA1 (transient receptor potential ankyrin 1) channels as a selective agonist. This action increases the influx of Ca^2+^ into the cell and raises its intracellular concentration. TRPA1 is a Ca^2+^-permeable non-selective cation channel in the plasma membrane that is believed to play an essential role in the development and progression of several cardiovascular diseases, including atherosclerosis, heart failure, ischemia-reperfusion injury, myocardial fibrosis, arrhythmia, and hypertension. The activation of TRPA1 has a protective role against the development of atherosclerosis, and this activation itself produces peripheral vasodilation and induces a biphasic blood pressure response. Conversely, a loss of expression of this channel or its blockade has been shown to suppress heart failure, ischemia-reperfusion injury of the myocardium, and arrhythmias; therefore, it is important to consider the potential dual effects of activating this channel, as well as the overall effects of AITC.

Through studying angiogenesis, Wu et al. [66] established that irisin stimulates the expression and activity of matrix metalloproteinases, specifically MMP-2 and MMP-9, in human umbilical vein endothelial cells (HUVECs). This stimulating effect of irisin on metalloproteinases was demonstrated in an *in vitro* model through mRNA analysis and protein expression analysis, as well as in an *in vivo* model using zebrafish. Increased MMP activity was confirmed using gelatin zymography, which assessed the capacity of MMPs to degrade gelatin. The authors also showed that the mechanism by which irisin acts on MMPs in endothelial cells is dependent on the ERK (extracellular signal-regulated kinase) signaling pathway, and a specific ERK inhibitor (U0126) successfully inhibited the irisin-induced enhanced activity of these enzymes. Although this study focused on the effect of irisin on endothelial cells and their angiogenesis, it is evident that irisin has the capacity to influence cardiovascular system cells as well. In this context, we can suggest that the statistically significant increase in irisin levels in the myocardia of female mice treated with an aqueous solution of sinigrin (Sinig._F_) compared to the control group of female mice (Cont._F_) could reflect the protective effect of irisin on the myocardium. This result correlates with the statistically significant increase in MMP-2 and MMP-9 levels in the myocardia of female mice treated with sinigrin compared to the control group (*p* ≤ 0.05). However, this result can be viewed from two perspectives. Matrix metalloproteinases (MMPs) are a family of zinc-dependent endopeptidases involved in various physiological and pathological processes, particularly those related to tissue remodeling, cellular differentiation, mobility, angiogenesis, cellular proliferation, migration, and apoptosis. Dysfunction of this family of proteins leads to various pathologies, tissue destruction, fibrosis, and matrix weakening [67]. From one viewpoint, elevated levels of MMP-2 and MMP-9, in light of the increased levels of irisin induced by sinigrin, could be interpreted as a reflection of the protective actions of both sinigrin and irisin in the myocardium, in the context of maintaining homeostasis and preventing cardiac hypertrophy and/or remodeling. Nonetheless, it should be noted that, unlike other studies that examine such effects on the heart using animal models of cardiac hypertrophy and related pathologies, our research focused on healthy experimental animals; therefore, we cannot draw clear conclusions regarding this protective effect. From another perspective, MMPs are predominant proteases responsible for the degradation of extracellular matrix proteins, and a balance between MMPs and their endogenous tissue inhibitors (TIMPs) is crucial for limiting extracellular matrix degradation [67]. The extracellular matrix is essential for maintaining vascular homeostasis and is thought to support stability and influence the behavior of vascular cells. MMPs can degrade extracellular matrix proteins, such as collagen or elastin, thereby disrupting the structural integrity of the vascular wall and leading to decreased elasticity [68]. MMPs and their tissue inhibitors have been shown to be important factors in acute and chronic heart failure, acute myocardial infarction, atherosclerosis, and cardiomyopathies. Elevated levels of MMP-2, TIMP-1, and the N-terminal peptide of type III collagen, as markers of extracellular matrix turnover in patients with acute heart failure, are considered a consequence of accelerated pathological remodeling of the heart [69]. Considering this, elevated levels of MMPs may also be interpreted as a reflection of potential toxic effects on the myocardium, especially given that our experimental animals did not exhibit cardiac hypertrophy, and the observed increase in MMP levels cannot be fully justified by “physiological turnover”. It should also be noted that, in the Sinig._F_ group, there was a statistically significant increase in the catalase and total glutathione levels compared to the Cont._F_ group, which may also indicate increased oxidative stress. Summarizing the overall results regarding the effect of sinigrin on molecular markers of the myocardium, we suppose that sinigrin leads to metabolic, inflammatory, and antioxidant changes that are more pronounced in female mice.

Although there are some noteworthy findings, this study has several limitations. Its limited sample size may reduce the statistical power of this study. Although long enough to assess toxic effects, the relatively short duration of this study is not sufficient to fully capture the long-term effects on the myocardium. While the sex-dependent effects are interesting and point towards potential hormonal influences, there is not enough information to draw conclusions about the underlying mechanisms. The use of pure aqueous sinigrin solution, rather than a more physiologically relevant delivery method (e.g., a diet with sinigrin-containing vegetables), may not accurately reflect sinigrin’s absorption and metabolism in natural settings. However, this is a general limitation of any study including glucosinolates, because of the immense diversity in the absorption and bioavailability of these compounds. This study presents interesting results, but uses only the ELISA method, which does not allow for making conclusions about the underlying mechanism of the results and demands cautious interpretations. This information may help guide future, larger studies to confirm or refute our hypotheses.

## 5. Conclusions

In conclusion, the results of this study indicate that the sub-chronic consumption of sinigrin may lead to a significant increase in the levels of the myokine irisin, matrix metalloproteinases (MMP-2 and MMP-9), catalase (CAT), and total glutathione (tGSH) in the myocardia of female mice. At the same time, there was a significant decrease in the levels of atrial natriuretic peptide (ANP) when compared to the control group of both female and male mice. Furthermore, the findings suggest that the hydrolysis products of sinigrin, after subchronic administration, may have a potentially toxic effect on the myocardia of female mice, as well as their ability to modulate transcription factors *in vivo* in a sex-dependent manner. This highlights the need for further research into the mechanisms of action of glucosinolates and their hydrolysis products, their interactions with sex hormones, and the permissible doses associated with both positive and negative effects, before implementing them as potential targeted therapeutics.

## Data Availability

The original contributions presented in this study are included in the article. Further inquiries can be directed to the corresponding author.
